# Validity and responsiveness of the EQ-5D in assessing and valuing health status in patients with anxiety disorders

**DOI:** 10.1186/1477-7525-8-47

**Published:** 2010-05-05

**Authors:** Hans-Helmut König, Anja Born, Oliver Günther, Herbert Matschinger, Sven Heinrich, Steffi G Riedel-Heller, Matthias C Angermeyer, Christiane Roick

**Affiliations:** 1 Department of Medical Sociology and Health Economics, University Medical Center Hamburg-Eppendorf, Martinistr. 52, 20246 Hamburg, Germany; 2 Health Economics Research Unit, Department of Psychiatry, University of Leipzig, Liebigstr. 26, D-04103 Leipzig, Germany; 3 Department of Medical Psychology and Medical Sociology, University of Leipzig, Philipp-Rosenthal-Str. 55, D-04103 Leipzig, Germany; 4 Department of Psychiatry, University of Leipzig, Semmelweisstr. 10, D-04103 Leipzig, Germany; 5 Department of Social Medicine, University of Leipzig, Philipp-Rosenthal-Str. 55, D-04103 Leipzig Germany; 6 Center for Public Mental Health, Untere Zeile 13, A-3482 Gösing am Wagram, Austria

## Abstract

**Background:**

The EQ-5D is a generic questionnaire which generates a health profile as well as index scores for health-related quality of life that may be used in cost-utility analysis.

**Aims of the study:**

To examine validity and responsiveness of the EQ-5D in patients with anxiety disorders.

**Methods:**

389 patients with anxiety disorders completed the EQ-5D at baseline and 6-month follow-up. Subjective measures of quality of life (WHOQOL-BREF) and psychopathology (BAI, BDI-II, BSQ, ACQ, MI) were used for comparison. Validity was analyzed by assessing associations between EQ-5D scores and related other scores. Responsiveness was analyzed by calculating effect sizes of differences in scores between baseline and follow-up for 3 groups indicating more, constant or less anxiety. Meaningful difference scores for shifting to less or more anxiety were derived by means of regression analysis.

**Results:**

88.4% of respondents reported problems in at least one of the EQ-5D dimension at baseline; the mean EQ VAS score was 63.8. The EQ-5D dimension most consistently associated with the measures used for comparison was 'anxiety/depression'. EQ VAS and EQ-5D index scores were highly correlated (|r|>0.5) with scores of the WHOQOL-BREF dimensions 'physical', 'mental' and 'overall' as well as BAI and BDI-II. The EQ-5D index tended to be the most responsive score. Standardized meaningful difference scores were not significantly different between EQ VAS, EQ-5D index and measures used for comparison.

**Conclusions:**

The EQ-5D seems to be reasonably valid and moderately responsive in patients with anxiety disorders. The EQ-5D index may be suitable for calculating QALYs in economic evaluation of health care interventions for patients with anxiety disorders.

**Trial registration:**

Current Controlled Trials ISRCTN15716049

## Background

The EQ-5D is a short generic patient-rated questionnaire for subjectively describing and valuing health-related quality of life (HRQOL); it is often used as an outcome measure in both clinical and health care services research (see complete list of references provided by the EuroQol Group [1]). The EQ-5D provides a descriptive profile of HRQOL as well as a subjective overall rating of the patient's own health state on the day of administration by means of a visual analogue scale (EQ VAS) [2,3]. Furthermore, according to a particular set of preference values derived from surveys of the general population, an index score (EQ-5D index) is assigned to all possible descriptive profiles of the EQ-5D. The EQ-5D index reflects the preferences of the community for EQ-5D health states. Preference-based scores are often used for calculating quality-adjusted life years (QALYs) in economic evaluation (cost-utility analysis) of health care [4,5]. According to a recent review, the EQ-5D is the most frequently used instrument to calculate QALYs [6].

Since decisions about the suitability of the EQ-5D in economic evaluation need to be based on a clear conceptual framework, the validity of the EQ-5D has been demonstrated for various diseases as well as the general population (see EuroQol Group's complete list of references). Although QALYs have been recommended as a measure of health effects in patient with anxiety disorders [7], and some evaluations of care for this patient group have already used the EQ-5D [8,9], little is known about the psychometric properties of the EQ-5D in patients with anxiety disorders: Supina et al. showed that the EQ-5D may be useful in distinguishing subjects with anxiety from mentally healthy subjects [10]. In a sample of patients with mood and/or anxiety disorders Lamers et al. found the EQ-5D index to capture improvements based on quartiles of the patient-rated Symptom Check List 90R (SCL-90R) [11]. Whynes et al. showed that the EQ-5D was responsive to differing degrees of anxiety identified by the Hospital Anxiety and Depression Scale's anxiety sub-scale in a sample of women with lowgrade cytological abnormalities detected at routine screening for cervical cancer [12].

The purpose of our study was to analyze the EQ-5D's psychometric properties in patients with anxiety disorders in more detail. More precisely, we analyzed its construct validity (Does the EQ-5D adequately measure the underlying construct?) as well as its responsiveness (Is the EQ-5D able to detect health state changes over time?).

## Methods

### Subjects and study design

Our study used a patient sample which was enrolled in a cluster randomised controlled trial (CRCT; trial registration: Current Controlled Trials ISRCTN15716049). For this trial, 8472 consecutive patients under treatment were screened for anxiety disorders with the German version of the Patient Health Questionnaire (PHQ-D) [13] at 54 practices of general practitioners (GPs) in the city area of Leipzig, Germany, from August to November 2005. 7.4% of the patients (n = 629) screened positive for panic disorder (F41.0), panic disorder with agoraphobia (F40.01), generalized anxiety disorder (F41.1) or unspecified anxiety disorder (F41.9) according to ICD-10 criteria [14]. Finally, 46 GPs participated in the CRCT, and 389 positively screened patients agreed to participate in the baseline assessment (t0) conducted from January to March 2006. Of the participating patients, 218 (56.0%) suffered from panic disorder, 114 (29.3%) from generalized anxiety disorder and 57 (14.7%) from unspecified anxiety disorder. After stratification of GP practices (clusters) by the number of patients, the clusters were randomly allocated to a control group (CG: 23 GP practices, n = 188 patients) and an intervention group (IG: 23 GP practices, n = 201 patients). GPs in the intervention group received training on diagnosis and treatment of anxiety disorders combined with a consultation offer during 6 months. After 6 months (t1), patients were re-assessed using the same set of measures described below. Drop-out rates were relatively low, resulting in 335 participating patients at t1 (CG: n = 163, IG: n = 172). Design and results of the CRCT have been reported in detail elsewhere [15]. As the intervention was not effective, with no differences between IG and CG in any outcome measure assessed, patients of the IG and CG were pooled for the analyses presented here. Socio-demographic characteristics of the sample at t0 are outlined in Table S1, Additional file 1.

The research protocol of the study was reviewed and approved by the Committee of Research Ethics at the Medical Faculty of the University of Leipzig.

### Measures

Besides the EQ-5D, all patients were assessed by a set of patient-rated questionnaires including the World Health Organization Quality of Life-Bref questionnaire (WHOQOL-BREF), the Beck Depression Inventory (BDI-II) as well as anxiety-specific measures of psychopathology (Beck Anxiety Inventory (BAI), Body Sensation Questionnaire (BSQ), Agoraphobic Cognitions Questionnaire (ACQ), and Mobility Inventory (MI)).

#### EQ-5D

The EQ-5D questionnaire comprises five questions (items) relating to current problems in the dimensions 'mobility', 'self-care', 'usual activities', 'pain/discomfort', and 'anxiety/depres-sion' [2,3,16]. Responses in each dimension are divided into three ordinal levels coded (1) no problems, (2) moderate problems, (3) extreme problems. This part, called the EQ-5D descriptive system, provides a five-dimensional description of health status which can be defined by a five-digit number. For example, the state '11122' indicates no problems in mobility, self-care, and usual activities, but moderate pain/discomfort and moderate anxiety/depression. Theoretically, 3^5 ^= 243 different health states can be defined. The EQ-5D descriptive system is followed by the EQ VAS, which is similar to a thermometer, ranging from 0 (worst imaginable health state) to 100 (best imaginable health state). The EQ VAS records the respondent's subjective overall rating of his/her own health state on the day of administration (EQ VAS score).

#### EQ-5D index

The EQ-5D index represents societal preference values for the full set of 243 EQ-5D health states with the state '11111' (perfect health) and 'death' a priori (i.e. by definition) being assigned values of 1 and 0, respectively. The EQ-5D index scores used in the present study were obtained from a random sample of the British general population (n = 2997), where the Time Trade-Off (TTO) method was used to derive preference values for given EQ-5D health states [17]. The British EQ-5D index has been used in numerous international studies, including the field of mental health [18]. Accordingly, to each patient's health status on the descriptive system of the EQ-5D, an index score was assigned, ranging from 1.0 for the EQ-5D health state 11111 to -0.59 for the worst possible health state (33333) [17].

#### WHOQOL-BREF

The WHOQOL-BREF is a generic questionnaire for subjectively measuring quality of life in the previous two weeks [19]. It is an abbreviated version of the WHOQOL-100 questionnaire which assesses a person's perception of their life situation in the context of their culture and value system and consists of 26 items, each rated on a 5-point Likert scale. Unlike other instruments, the WHOQOL-BREF takes a broader concept of quality of life, which is not restricted to health-related aspects. Since numerous international centers participated in the development of the WHOQOL it is a culturally independent instrument. From the 26 items, scores for four domains can be derived, namely 'physical health' (7 items), 'mental health' (6 items), 'social relationships' (3 items) and 'environment' (8 items). In addition, the WHOQOL-BREF provides a score for overall quality of life, based on two items. All scores range from 1 (worst) to 100 (best). Domain and overall scores were calculated according to the scoring algorithms provided by the manual of the German version of the WHOQOL-BREF. The psychometric properties of the WHOQOL-BREF are considered good with an internal consistency (Cronbach's α) of the subscales between 0.76 and 0.88. The validity has been established through a good discriminative ability between healthy and ill subjects [20].

#### BAI

The BAI is a 21-item measure designed to assess the severity of self-reported anxiety [21]. Responses on each item range from 0 (*not at all bothered*) to 3 (*severely bothered*). The total score for all 21 items ranges from 0 to 63, with higher scores indicating higher levels of anxiousness. The BAI has excellent internal consistency (Cronbach's α = 0.93) [22] and good test-retest reliability (r = 0.73 to 0.83) [21,23,24]. Convergent validity in clinical samples was r = 0.51 with the Hamilton Anxiety Scale [21], r = 0.58 with the State-Trait-Anxiety-Inventory (STAI) Trait and r = 0.47 with the STAI State [24]. Its discriminant validity was superior that of the STAI [24]. The BAI is one of the most commonly used measures to assess the construct of anxiety [25], because it is easy to administer, to complete, and to interpret.

#### BDI-II

The BDI-II is a 21-item self-report depression screening measure [26]. The items ask respondents to endorse statements characterizing how they have been feeling throughout the last week. Here each item is rated on a scale ranging from 0 to 3, with a possible range of total scores from 0 to 63. Higher scores represent a higher intensity level of depression. Scores of 0 to 13 denote minimal depression, scores of 14 to 19 denote mild depression, scores of 20 to 28 denote moderate depression, and scores of 29 to 63 denote severe depression.

#### BSQ

The BSQ is 17-item self-report instrument to evaluate fear of the physical sensations generally associated with a panic attack [27]. The BSQ has very good internal consistency (Cronbach's α = 0.87) and moderate test-retest reliability (r = 0.67). It showed good construct validity with a variety of measures of psychopathology.

#### ACQ

The ACQ is a 14-item self-report instrument which measures the frequency of fearful cognitions associated with panic attacks and agoraphobia [27]. In a sample of outpatients with agoraphobia, internal consistency measured by Cronbach's α was 0.80.

#### MI

The MI is a 29-item self-report instrument measuring the severity of behavioral avoidance [28,29]. Internal consistency measured by Cronbach's α ranged from α = 0.91 to 0.97, test-retest reliability ranged from *r *= 0.75 to 0.90, and good construct validity has been shown. The MI is divided into two subscales, Avoidance Alone (MIA) and Avoidance Accompanied (MIB), with good internal consistency for both subscales.

Scores of BSQ, ACQ, MIA and MIB each may range from 0 (best) to 4 (worst). German versions of these instruments are described in [30].

### Psychometric analysis

The EQ-5D is a generic instrument intended to measure overall HRQOL in a wide range of populations, including patients with anxiety disorders. Yet, the extent to which the EQ-5D is successful in measuring HRQOL in patients with anxiety disorders has not been examined in depth. Therefore the approach taken in this study was to compare the EQ-5D with other measures of psychopathology and quality of life. Thereby we assessed aspects of the EQ-5D's construct validity and responsiveness.

Construct validity refers to the extent to which the measurement corresponds to theoretical concepts (constructs) concerning the phenomenon under study [31]. In this study we hypothesized that there was an association between the scores of the EQ-5D and of measures of psychopathology and quality of life. While most applied measures of psychopathology specifically aim at measuring anxiety, the WHOQOL-BREF aims at measuring overall HRQOL similar to the EQ-5D, yet in a more detailed manner with the 'mental health' domain score being based on six items.

Thus, construct validity of the EQ-5D descriptive system was assessed by analysing the association between the response level of EQ-5D items and the scores of the other measures used in the study. Specifically, we hypothesized that the association was particularly strong between the EQ-5D dimension 'anxiety/depression' and the anxiety-specific BAI score as well as the mental health domain score of the WHOQOL-BREF. Construct validity of the EQ VAS and the EQ-5D index was assessed by analysing their correlation with the scores of the other measures used in the study. Specifically, we hypothesized that EQ VAS and EQ-5D index scores were strongly correlated with the BAI score and the mental health domain score of the WHOQOL-BREF.

Responsiveness was assessed by the criteria of the United States Food and Drug Administration (FDA), which raised a draft on patient reported outcomes including methods to calculate responsiveness and to interpret the detected changes as meaningful [32]. The recommended best practice in the evaluation of responsiveness is the calculation of various distribution-based estimates (i.e. effect size, standardized response mean) under several anchor-based criteria (i.e. patient or clinician ratings of global improvement) [33]: on this note, the anchor-based criterion is used as an external indicator to assign patients into groups reflecting 'no change' and a '(small) positive/negative change' [34,35]. Guidance on interpretation of the magnitude of a distribution-based estimate, for example, whether differences in scores are viewed as meaningful, is provided [36,37]. Nonetheless, there is no gold standard in terms of whether the difference in scores is meaningful, but there are some methods which one will find very useful in interpretation; for example, one definition of a meaningful difference is based on the 'minimal important difference' (MID) [34,35]. In practice, the MID is viewed as the difference in scores between the group with 'no change' and the group with 'small positive/negative change'.

### Data analytic procedures

The non-parametric Spearman rank correlation coefficient (r_s_) was used for analysing correlations, since the EQ VAS score and the EQ-5D index were not normally distributed. Correlation was considered *large *for |r_s_|≥0.5, *moderate *for 0.3≤|r_s_|<0.5, and *small *for 0.1≤|r_s_|<0.3 [36]. Responsiveness was compared by paired t-test statistics, effect sizes (ES) and standardized response mean (SRM). The method to calculate ES was: ES = M_1_-M_0_/SD_baseline_; where M_0 _is the mean score of the baseline assessment, M_1 _the mean score of the follow-up assessment, and SD_baseline _the standard deviation of the baseline assessment. The method to calculate SRM was: SRM = M_1_-M_0_/SD_M1-M0_; where the numerator remains the same as for calculating ES, but the denominator represents the SD of the difference in scores. We considered an absolute magnitude of difference scores expressed by ES and SRM <|0.20| as trivial, from ≥|0.20| to <|0.50| as small, from ≥|0.50| to <|0.80| as medium, and ≥|0.80| as large based on Cohen's interpretation guidelines [36]. Meaningful differences in health status were estimated as follows [32]: Patients whose psychopathology on the BAI increased by more than 0.5 SD of the BAI baseline score from t0 to t1 were categorized into a group labelled 'more anxiety' (*n *= 43). Patients whose BAI score decreased by more than 0.5 SD were categorized into a group labelled 'less anxiety' (*n *= 83). All other patients were categorized as 'no shift in anxiety' (*n *= 124). Categorizing of patients was performed independently of any intervention. Five linear regression models with the difference scores of EQ VAS, EQ-5D index, WHOQOL-BREF domain score 'mental health', BSQ and ACQ used as dependent variables were estimated. Each dependent variable was regressed on dummy variables for 'more anxiety', 'less anxiety' as well as respondents' age, the BDI-II score and the score of the respective measure at baseline. The absolute values of coefficients of the dummy variables were tested for significant differences within each regression model. For better comparison between the models, difference scores and baseline scores were standardized to SD = 1 and mean = 0. Cross-model hypotheses were tested using seemingly unrelated estimation (SUEST) [38]. Statistical significance of raised R^2 ^due to incorporation of the anchor was tested using LR-tests with hierarchical nested regression models. For statistical testing, the level of significance was set at α = 0.05. Calculations were performed using the software package STATA [39].

## Results

### EQ-5D scores

**Figure 1:**
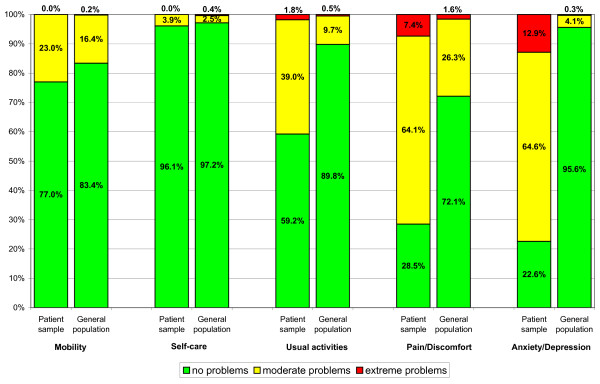
**Distribution of responses to items of EQ-5D descriptive system in patient sample (n = 389) and general population (n = 3552) **[40]. In the patient sample there were 7 missing values for the EQ-5D items 'mobility', 'self-care', 'usual activities', 10 missing values for the item 'pain/discomfort' and 8 missing values for the item 'anxiety/depression'.

Problems in at least one of the EQ-5D dimension were reported by 88.4% of the respondents at baseline. Most frequent were problems in the dimension 'anxiety/depression' (77.4%), followed by 'pain/discomfort' (71.5%), 'usual activities' (40.8%), 'mobility' (23.0%) and 'self-care' (3.9%). In all dimensions problems were reported more frequently than in a German general population sample [40] (Figure 1).

The most frequently reported EQ-5D health state was the one with moderate problems in the two dimensions "pain/discomfort" and "anxiety/depression" (11122), which was indicated by 21.2% of all respondents (Table S2, Additional file 2). The four EQ-5D health states reported most frequently covered 59.4% of all respondents.

The mean (median) was 63.8 (70) for the EQ VAS score and 0.66 (0.73) for the EQ-5D index (Table S3, Additional file 3). The proportion of respondents with the best possible score was 1.0% for the EQ VAS and 11.6% for the EQ-5D.

### Score of measures used for comparison

Table S4, Additional file 4 presents the scores of the measures used for comparison with the EQ-5D at baseline. The mean scores of WHOQOL-BREF domains 'physical health', 'social relationships' and 'environment' were around 60 and those of 'mental health' and the 'overall' score were around 50. The mean BAI score was 20.4: the mean BDI-II score (16.4) indicated mild depression on average.

### Construct validity

Table S5, Additional file 5 shows the mean scores of the measures used for comparison categorized by the patients' level of response to EQ-5D items at baseline. Since for all EQ-5D items the number of observations in the category 'extreme problems' was small, the categories 'moderate problems' and 'extreme problems' were collapsed into one category.

For almost all EQ-5D dimensions, different response levels were associated with significantly different scores of the WHOQOL-BREF domains and measures of psychopathology. As hypothesized, the strongest association was found between the EQ-5D dimension 'anxiety/depression' and the BAI score: For respondents reporting moderate or extreme problems in the EQ-5D dimension 'anxiety/depression', the mean BAI score was 23.7, whereas for those reporting no problems in this dimension it was 9.2. This difference was highly significant and corresponds to an ES of *d *= 1.53. Also, as hypothesized, a very strong association of the EQ-5D dimension 'anxiety/depression' with the 'mental health' domain score of the WHOQOL-BREF (*d *= 1.44) was found, but also with the BDI-II (*d *= 1.52). ES of all other measures were smaller, yet still large (|d|>0.8), except for the WHOQOL-BREF domain score 'environment' and the MI scores. For different levels of all other EQ-5D dimensions, ES of the WHOQOL-BREF domain score 'physical health' were largest (always |d|>0.8).

The WHOQOL-BREF domain score 'physical health' and the 'overall' score showed large ES for different levels of all EQ-5D dimensions, whereas the ES of the WHOQOL-BREF domain score 'mental health' was only large for different levels of the EQ-5D dimensions 'self-care' and 'anxiety/depression'. The scores of BAI and BDI-II showed large ES for different levels of the EQ-5D dimensions 'anxiety/depression', 'usual activities' and 'self-care'. The scores of BSQ and ACQ showed large ES only for different levels of the EQ-5D dimension 'anxiety/depression', whereas the scores MIA and MIB did not show any large ES.

Regarding the construct validity of the EQ VAS score and EQ-5D index, Table S6, Additional file 6 shows large correlations with the BAI and the WHOQOL-BREF domain 'mental health', as hypothesized. Yet, correlation was strongest with the WHOQOL-BREF domain 'physical health', and also large with the BDI-II and the WHOQOL-BREF 'overall' score. All correlations of the EQ VAS score and the EQ-5D index with scores of BSQ, ACQ, MIA and MIB were weaker and only moderate.

### Responsiveness of the EQ VAS and the EQ-5D indices compared with other measures

Table S7, Additional file 7 shows the responsiveness statistics (t-statistics, ES and SRM) for all outcome measures, split by the anchor of change defined by the BAI score. While the t-statistics indicated significant differences in scores of all outcome measures for patients who shifted to 'more anxiety' or 'less anxiety', large ES were only observed for the EQ-5D index in the category "more anxiety". For all other measures, ES were mostly trivial to medium in the categories "more anxiety" and "less anxiety". SRM were never larger, but some were medium, especially in the category 'more anxiety'. In the category 'constant anxiety', ES and SRM were negligible for all outcome measures, and t-statistics indicated no significant differences.

### Meaningful differences of the EQ VAS and the EQ-5D index compared with other measures

Table S8, Additional file 8 presents the results of the five regression models. Regression coefficients of the dummy variables representing a shift to 'more anxiety' were all significant and tended to have larger absolute values than those representing a shift to 'less anxiety', which were not significant for the WHOQOL-BREF-mental domain score. The EQ-5D index indicated a significant gain of 0.07 score points (standardized: 0.29) as a meaningful difference associated with a shift to 'less anxiety' and a significant reduction of -0.15 (standardized: -0.67) as a meaningful difference associated with a shift to 'more anxiety'. The effects of all measures' baseline scores were significant and negative indicating larger differences between the two assessments with smaller scores at the baseline. Co-morbid depression measured by the BDI-II did not affect meaningful difference scores of any of the measures except for a small effect on the BSQ meaningful difference score.

When testing intra-model hypotheses, the difference scores in the categories 'less anxiety' and 'more anxiety' were significantly different for all outcome measures, whereas the absolute values of difference scores in both categories were not significantly different. The inclusion of the dummy variables for 'more anxiety' and 'less anxiety' resulted in a significant increase of the R^2 ^in all six models. When testing cross-model hypotheses with SUEST, the effects (coefficient values) of 'more anxiety' (*P *= 0.30), 'less anxiety' (*P *= 0.42) and 'constant anxiety' (*P *= 0.30) on standardized outcome measures were not significantly different in the five regression models. Effects of standardized baseline scores differed significantly across the six outcome measure (*P *= 0.03).

## Discussion

Although the EQ-5D has been used in patients with anxiety disorders before [8,9], little data on its psychometric properties in this patient group have been published [10-12].

Patients with anxiety disorders were mostly burdened in the EQ-5D dimensions 'anxiety/depression', 'pain/discomfort', and 'usual activities'. Only 11.4% of respondents reported no problems in any of the EQ-5D dimensions, and only 1.0% rated their health states with the best possible score (100) on the EQ VAS. Thus, the ceiling effect of the EQ-5D which has been described repeatedly in other patient groups [41] as well as in samples of the general population [42] seems to be less pronounced in this patient group. The EQ-5D discriminated well between patients with anxiety disorders and the general population as anxiety patients reported problems in all dimensions more frequently. This goes in line with findings of Supina et al. who showed increased odds of anxiety patients for reporting problems in all EQ-5D dimensions compared to mentally healthy subjects [10].

The EQ-5D dimension most consistently associated with the subjective measures of psychopathology and quality of life was 'anxiety/depression' followed by the dimensions 'self-care' and 'usual activities'. As hypothesized, the strongest association was found between the EQ-5D dimension 'anxiety/depression' and the BAI score. This is indicative of an adequate construct validity of the EQ-5D. This is also emphasized by the strong correlation of EQ VAS and EQ-5D index scores with the BAI score and the 'mental health' domain score of the WHOQOL-BREF. Not surprisingly, association with the BDI-II was also strong, as the EQ-5D does not differentiate between anxiety and depression. Nevertheless, we would have expected to find larger ES and stronger correlations with the BSQ and ACQ since these measures are considered to be more sensitive than the BAI. Yet being a generic HRQOL instrument designed to measure problems in all major dimensions of well-being, the EQ-5D inevitably has only limited content validity for specific diseases. Only one out of five questions of the EQ-5D specifically refers to anxiety or mental health.

The EQ-5D index tended to be more responsive than the EQ VAS score, showing larger ES and SRM and significant coefficients associated with both, shifts to more and less anxiety in the regression model analyzing meaningful differences. However, when comparing regression coefficients across the five models using SUEST, no significant differences in meaningful differences between the various measures were found, pointing at similar responsiveness of the EQ-5D and the measures used for comparison.

Although the EQ-5D index seemed more responsive to the shift to *more *anxiety than to the shift to *less *anxiety statistical testing indicated that, while significantly different, both response levels did not differ significantly in their absolute values. Therefore, greater responsiveness of EQ-5D scores for a shift to 'worse health status' than for a shift to 'better health status' as reported in a study by Günther et al. [43] in patients with depression could not be confirmed. One possible explanation might be the less pronounced ceiling effect of the EQ-5D in anxiety patients. It was argued by Günther [43] that, as a consequence of a ceiling effect, the larger range at the bottom of the EQ-5D indices could provide a potential for the assessment of larger changes in health state than at the compressed top of the scale. An alternative explanation might be that our finding is the result of adequate testing.

The 'physical health' domain score of the WHOQOL-BREF showed strong association with all EQ-5D dimensions as well as strong correlation with EQ VAS and EQ-5D index scores. Changes in anxiety defined the BAI score were often associated with changes in problem levels of EQ-5D dimensions other than 'anxiety/depression'. This indicates the important role of psychosomatic aspects and somatic comorbidity in patients with anxiety disorders. Unfortunately, we did not specifically assess co-morbid somatic conditions in our study. Therefore responsiveness statistics and estimates for meaningful difference scores might be confounded by somatic co-morbidity which is a limitation of our study. However, controlling for mental co-morbidity measured by the BDI-II hardly affected meaningful difference scores. Yet, somatic co-morbidity may explain the conspicuously large ES of the EQ-5D index in the group with more anxiety. In this small group (n = 43), changes in the EQ-5D index were largely due changes in the EQ-5D dimension 'anxiety/depression' and/or 'mobility', the latter possibly caused by somatic co-morbidity. However, the fact that the corresponding SRM is much smaller (due to a large SD of the change score of the EQ-5D) points at the presence of outliers for which this small patient group is more sensitive than the larger groups with constant or more anxiety.

A limitation of this study results from including patients based on the results of a screening instrument (PHQ-D) and not on formal diagnosis of an anxiety disorder. As the PHQ has a high specificity (97%) [44], probably most of the study participants met formal criteria for anxiety disorders. The mean score of the BAI at baseline was similar to mean BAI scores found in other samples of outpatients with anxiety disorders [21,24]. On the other hand, the comparatively low sensitivity of the PHQ (67%) [44] resulted in approximately one third of patients with anxiety disorders being missed, which corresponds to the prevalence of 7.6% found in our primary care sample as opposed to higher prevalence rates of anxiety found in other primary care samples [45].

Although the use of country-specific EQ-5D index scores have been recommended, we used the British EQ-5D index [17] instead of the German index reported by Greiner et al. [46]. The German EQ-5D index was estimated based on a rather small population sample (N = 334) and must therefore be considered less precise: German index scores for all 243 EQ-5D health states were estimated from a sample of 36 states using a regression model. In particular, scores predicted by the model for EQ-5D health states which are frequent in patients with anxiety disorders such as 11112 (German EQ-5D index = 1.0) were much higher than those measured in the general population sample due to omitted regression coefficients. Thus, German EQ-5D index scores must be considered preliminary. A further limitation results from collapsing the levels "extreme problems" and "moderate problems" into one category. As mentioned earlier, the combining of levels was done for statistical reasons and resulted from the extremely low number of responses but might have limited the discriminative ability of the EQ-5D.

Analyzing the validity of the EQ-5D by determining its relationship with the measures used for comparison might be problematic, since these measures might lack validity for themselves. Yet, psychometric properties reported in the literature of BAI, BSQ, ACQ and MI for patients with anxiety disorders appear to be good. However, we used only patient-rated but no clinician-rated measures for comparison, which also limits the analysis of validity.

Moreover, the responsiveness analysis might have suffered from the absence of a practical clinical criterion. For defining the anchors we used the BAI score which, because of its excellent psychometric properties, good convergent validity and better discriminant validity than other measures of anxiety, is the most commonly measure to assess the construct of anxiety. However, the anchors were built by a rather statistical approach which does not necessarily imply "meaningfulness". Yet, the statistical significance of the EQ-5D's responsiveness is clearly indicated by significant increase of R^2 ^when the BAI-anchor was included in the regression analysis.

Due to differences in the applied measures for comparison and in statistical methods, our results are difficult to compare with other studies which have analyzed the psychometric properties of the EQ-5D in patients with psychiatric disorders. Yet, other studies reported slightly larger correlations of EQ VAS and EQ-5D index scores with the score of the WHOQOL-BREF-mental domain in alcohol dependent patients (EQ VAS: r = 0.55; EQ-5D index: r = 0.60) [47] and in patients with schizophrenia (0.62; 0.58) [48] than our study (0.51; 0.50). Change scores of EQ VAS and EQ-5D index associated with changed likelihood of HADS-defined anxiety reported by Whynes et al. [12] are similar in magnitude to meaningful difference scores found in our study. However, meaningful difference scores of the EQ VAS and the EQ-5D index estimated in a sample of patients with depression tended to be larger, yet based on different anchors [43]. Thus, construct validity and responsiveness of the EQ-5D in patients with anxiety disorders might be somewhat worse than in other psychiatric patients.

## Conclusions

The EQ-5D appears to be a reasonably valid instrument with moderate responsiveness in patients with anxiety disorders. Being a short and easy to administer questionnaire, the EQ-5D may easily be added to disorder-specific measures, when overall HRQOL and its valuation are considered important outcomes. The EQ-5D index may be suitable for calculating QALYs in economic evaluation of health care interventions for patients with anxiety disorders. Future studies should also use clinician-rated measures for analyzing validity and responsiveness.

## Competing interests

The authors declare that they have no competing interests.

## Authors' contributions

HHK designed the study, wrote the protocol, and wrote the first draft of the manuscript. AB conducted the field work. HM undertook the statistical analyses. OG, AB, SGRH, MCA, SH and CR designed the study, wrote the protocol and managed the field work. All authors contributed to and have approved the final manuscript.

## Supplementary Material

Additional file 1 **Table S1**. Socio-demographic sample characteristics at baseline (N = 389)^a^ Click here for file

Additional file 2 **Table S2**. The 10 most frequently reported EQ-5D health states at baseline (N = 372)^a^ Click here for file

Additional file 3 **Table S3**. Descriptives of the EQ VAS score and the EQ-5D index at baseline^a^ Click here for file

Additional file 4 **Table S4**. Score of measures used for comparison at baseline Click here for file

Additional file 5 **Table S5**. Association between response level of EQ-5D items and score of other measures at baseline^a^ Click here for file

Additional file 6 **Table S6**. Correlation between EQ VAS score, EQ-5D index and scores of other measures at baseline^a^ Click here for file

Additional file 7 **Table S7**. Comparison of responsiveness statistics for EQ-5D scores and scores of measures used for comparison by anchor of change defined by BAI score Click here for file

Additional file 8 **Table S8**. Results of the regression models estimating meaningful difference scores for various measures according to the anchor defined by the BAI score^a^, controlled for measure's baseline score, BDI-II score and age of respondent (N = 230)^b^ Click here for file
