# Supplementary material for: Validity and responsiveness of the EQ-5D in assessing and valuing health status in patients with anxiety disorders
Source: Health Qual Life Outcomes. 2010 May 5;8:47. doi: 10.1186/1477-7525-8-47 (PMC2873595; doi:10.1186/1477-7525-8-47)
Supplement: Additional file 1 — Table S1. Socio-demographic sample characteristics at baseline (N = 389)a [file 1477-7525-8-47-S1.DOC]

**Table S1.** Socio-demographic sample characteristics at baseline (N=389)^a^

| Characteristics | N | % |
| --- | --- | --- |
| Gender |  |  |
| Male | 99 | 25.52 |
| Female | 289 | 74.48 |
| Family status |  |  |
| Single | 157 | 40.36 |
| Married | 157 | 40.36 |
| Divorced | 68 | 17.48 |
| Widowed | 7 | 1.80 |
| Living situation |  |  |
| Living alone | 120 | 30.93 |
| With spouse/partner | 228 | 58.76 |
| With relatives | 18 | 4.64 |
| Other | 22 | 5.67 |
| Education |  |  |
| Low | 65 | 16.71 |
| Middle | 207 | 53.21 |
| High | 117 | 30.08 |
| Age: Mean, SD | 41.69 | 12.10 |

**^a^** Number of observations n<389 for some variables is due to missing values.
