# Supplementary material for: Validity and responsiveness of the EQ-5D in assessing and valuing health status in patients with anxiety disorders
Source: Health Qual Life Outcomes. 2010 May 5;8:47. doi: 10.1186/1477-7525-8-47 (PMC2873595; doi:10.1186/1477-7525-8-47)
Supplement: Additional file 2 — Table S2. The 10 most frequently reported EQ-5D health states at baseline (N = 372)a [file 1477-7525-8-47-S2.DOC]

Table S2. The 10 most frequently reported EQ-5D health states at baseline (N=372)^a^

| EQ-5D health state | n (%) |
| --- | --- |
| 11122 | 79 (21.2) |
| 11112 | 51 (13.7) |
| 11222 | 48 (12.9) |
| 11111 | 43 (11.6) |
| 21222 | 28 (7.5) |
| 11121 | 27 (7.3) |
| 11223 | 12 (3.2) |
| 11123 | 9 (2.4) |
| 21223 | 9 (2.4) |
| 21232 | 8 (2.2) |

^a^ This table lists all health states which were reported at least 8 times among all respondents. Hence, percentages do not sum up to 100%.
