# Supplementary material for: Validity and responsiveness of the EQ-5D in assessing and valuing health status in patients with anxiety disorders
Source: Health Qual Life Outcomes. 2010 May 5;8:47. doi: 10.1186/1477-7525-8-47 (PMC2873595; doi:10.1186/1477-7525-8-47)
Supplement: Additional file 3 — Table S3. Descriptives of the EQ VAS score and the EQ-5D index at baselinea [file 1477-7525-8-47-S3.DOC]

**Table S3.** Descriptives of the EQ VAS score and the EQ-5D index at baseline^a^

|  | EQ VAS score | EQ-5D index |
| --- | --- | --- |
| Mean | 63.8 | 0.662 |
| Standard deviation | 20.8 | 0.269 |
| Range | 0 - 100 | -0.24 - 1.00 |
| Percentiles |  |  |
| 90 | 90 | 1.000 |
| 75 | 80 | 0.848 |
| 50 | 70 | 0.725 |
| 25 | 50 | 0.620 |
| 10 | 35 | 0.197 |
| n of max. score (%) | 4 (1.05) | 43 (11.56) |

**^a^** The number of observations was 382 for the EQ VAS score and 372 for the EQ-5D index due to missing values
