# Supplementary material for: Validity and responsiveness of the EQ-5D in assessing and valuing health status in patients with anxiety disorders
Source: Health Qual Life Outcomes. 2010 May 5;8:47. doi: 10.1186/1477-7525-8-47 (PMC2873595; doi:10.1186/1477-7525-8-47)
Supplement: Additional file 4 — Table S4. Score of measures used for comparison at baseline [file 1477-7525-8-47-S4.DOC]

Table S4. Score of measures used for comparison at baseline

| Measures | Possible range of score (worst-best) | N**^a^** | Score | |
| --- | --- | --- | --- | --- |
|  |  |  | Mean (SD) | Median (range) |
| WHOQOL-BREF |  |  |  |  |
| Physical health | (0-100) | 384 | 59.1 (19.1) | 60.7 (7.1-100) |
| Mental health | (0-100) | 384 | 50.7 (19.2) | 50.0 (0.0-100) |
| Social relationships | (0-100) | 388 | 56.9 (22.2) | 58.3 (0.0-100) |
| Environment | (0-100) | 381 | 60.2 (14.5) | 62.5 (12.5-96.9) |
| Overall | (0-100) | 387 | 48.4 (19.0) | 50.0 (0.0-100) |
| BAI | (63-0) | 326 | 20.4 (11.8) | 20.0 (0.0-55.0) |
| BDI-II | (63-0) | 344 | 16.4 (10.1) | 15.5 (0.0-48.0) |
| BSQ | (4-0) | 380 | 1.1 (0.7) | 1.0 (0.0-3.5) |
| ACQ | (4-0) | 385 | 0.7 (0.5) | 0.6 (0.0-3.0) |
| MIA | (4-0) | 350 | 0.7 (0.7) | 0.3 (0.0-3.4) |
| MIB | (4-0) | 357 | 1.1 (1.0) | 0.8 (0.0-4.0) |

^a^ Number of observations n<389 is due to missing values; WHOQOL-BREF, World Health Organization Quality of Life-Bref questionnaire; BAI, Beck Anxiety Inventory; BDI-II, Beck Depression Inventory; BSQ, Body Sensation Questionnaire, ACQ, Agoraphobic Cognitions Questionnaire; MIA, Mobility Inventory - Subscale Avoidance Alone; MIB, Mobility Inventory - Subscale Avoidance Accompanied.
