# Supplementary material for: Validity and responsiveness of the EQ-5D in assessing and valuing health status in patients with anxiety disorders
Source: Health Qual Life Outcomes. 2010 May 5;8:47. doi: 10.1186/1477-7525-8-47 (PMC2873595; doi:10.1186/1477-7525-8-47)
Supplement: Additional file 5 — Table S5. Association between response level of EQ-5D items and score of other measures at baselinea [file 1477-7525-8-47-S5.DOC]

Table S5. Association between response level of EQ-5D items and score of other measures at baseline^a^

|  | Mean scores of other measures by response level of EQ-5D items | | | | | | | | | | | | | | |
| --- | --- | --- | --- | --- | --- | --- | --- | --- | --- | --- | --- | --- | --- | --- | --- |
| EQ-5D item: | Mobility | | | Self-Care | | | Usual Activities | | | Pain/Discomfort | | | Anxiety/Depression | | |
| Problems: | no | yes | d^b^ | no | yes | d^b^ | no | yes | d^b^ | no | yes | d^b^ | no | yes | d^b^ |
| WHOQOL-BREF |  |  |  |  |  |  |  |  |  |  |  |  |  |  |  |
| Physical health | 64.2 ^3^ | 40.3 ^3^ | **1.48** | 59.9 ^3^ | 35.8 ^3^ | **1.47** | 68.3 ^3^ | 45.1 ^3^ | **1.49** | 72.4 ^3^ | 53.6 ^3^ | **1.12** | 74.4 ^3^ | 54.5 ^3^ | **1.22** |
| Mental health | 52.8 ^3^ | 42.7 ^3^ | 0.56 | 51.1 ^2^ | 37.6 ^2^ | **0.82** | 55.9 ^3^ | 43.0 ^3^ | 0.72 | 58.2 ^3^ | 47.5 ^3^ | 0.56 | 68.9 ^3^ | 45.3 ^3^ | **1.44** |
| Social relationships | 58.1 ^1^ | 52.3 ^1^ | 0.26 | 57.1 | 52.8 | 0.19 | 59.8 ^2^ | 52.9 ^2^ | 0.31 | 61.7 ^2^ | 54.9 ^2^ | 0.31 | 71.0 ^3^ | 52.7 ^3^ | **0.95** |
| Environment | 61.7 ^3^ | 55.1 ^3^ | 0.45 | 60.6 ^1^ | 50.9 ^1^ | 0.63 | 63.0 ^3^ | 56.3 ^3^ | 0.47 | 64.5 ^3^ | 58.6 ^3^ | 0.42 | 67.7 ^3^ | 58.1 ^3^ | 0.71 |
| Overall | 52.1 ^3^ | 35.5 ^3^ | **0.94** | 49.3 ^3^ | 25.8 ^3^ | **1.35** | 56.2 ^3^ | 37.0 ^3^ | **1.15** | 59.9 ^3^ | 43.8 ^3^ | **0.92** | 62.5 ^3^ | 44.2 ^3^ | **1.11** |
| BAI | 19.1 ^3^ | 25.7 ^3^ | 0.56 | 20.2 ^2^ | 32.0 ^2^ | **0.90** | 16.8 ^3^ | 26.1 ^3^ | **0.84** | 15.3 ^3^ | 22.9 ^3^ | 0.68 | 9.2 ^3^ | 23.7 ^3^ | **1.53** |
| BDI-II | 15.0 ^3^ | 21.6 ^3^ | 0.68 | 16.2 ^2^ | 25.0 ^2^ | **0.83** | 13.5 ^3^ | 21.1 ^3^ | **0.80** | 12.1 ^3^ | 18.4 ^3^ | 0.64 | 7.0 ^3^ | 19.3 ^3^ | **1.52** |
| BSQ | 2.0 ^3^ | 2.3 ^3^ | 0.45 | 2.1 | 2.4 | 0.41 | 1.9 ^3^ | 2.4 ^3^ | 0.65 | 1.8 ^3^ | 2.2 ^3^ | 0.63 | 1.6 ^3^ | 2.2 ^3^ | **1.00** |
| ACQ | 1.7 | 1.7 | 0.11 | 1.7 | 1.8 | 0.23 | 1.6 ^3^ | 1.8 ^3^ | 0.36 | 1.6 ^2^ | 1.7 ^2^ | 0.32 | 1.4 ^3^ | 1.8 ^3^ | **0.89** |
| MIA | 1.6 ^3^ | 2.0 ^3^ | 0.53 | 1.7 | 1.9 | 0.21 | 1.5 ^3^ | 2.0 ^3^ | 0.70 | 1.4 ^3^ | 1.8 ^3^ | 0.62 | 1.4 ^3^ | 1.7 ^3^ | 0.46 |
| MIB | 2.0 ^3^ | 2.5 ^3^ | 0.50 | 2.1 | 1.9 | 0.16 | 1.9 ^3^ | 2.4 ^3^ | 0.63 | 1.7 ^3^ | 2.3 ^3^ | 0.60 | 1.6 ^3^ | 2.2 ^3^ | 0.78 |

^a^There were missing values for the score of some of the other measures used for comparison, the number of observations is presented in Table 4; ^b^d=effect size (Cohen’s d), large effect sizes (|d|≥0.8) are printed bold; p-value for difference in mean score by response level of EQ-5D item: ^1^ p<0.05; ^2^ p<0.01; ^3^ p<0.001; WHOQOL-BREF, World Health Organization Quality of Life-Bref questionnaire; BAI, Beck Anxiety Inventory; BDI-II, Beck Depression Inventory; BSQ, Body Sensation Questionnaire, ACQ, Agoraphobic Cognitions Questionnaire; MIA, Mobility Inventory - Subscale Avoidance Alone; MIB, Mobility Inventory - Subscale Avoidance Accompanied.
