# Supplementary material for: Validity and responsiveness of the EQ-5D in assessing and valuing health status in patients with anxiety disorders
Source: Health Qual Life Outcomes. 2010 May 5;8:47. doi: 10.1186/1477-7525-8-47 (PMC2873595; doi:10.1186/1477-7525-8-47)
Supplement: Additional file 6 — Table S6. Correlation between EQ VAS score, EQ-5D index and scores of other measures at baselinea [file 1477-7525-8-47-S6.DOC]

Table S6. Correlation between EQ VAS score, EQ-5D index and scores of other measures at baseline^a^

| Measures | Correlation EQ VAS score | Correlation EQ-5D index |
| --- | --- | --- |
| WHOQOL-BREF |  |  |
| Physical health | **0.71** | **0.70** |
| Mental health | **0.51** | **0.50** |
| Social relationships | 0.25 | 0.26 |
| Environment | 0.33 | 0.33 |
| Overall | **0.65** | **0.58** |
| BAI | **-0.50** | **-0.53** |
| BDI-II | **-0.59** | **-0.54** |
| BSQ | -0.39 | -0.40 |
| ACQ | -0.29 | -0.32 |
| MIA | -0.37 | -0.35 |
| MIB | -0.35 | -0.36 |

^a^ Spearman rank correlation coefficient; all correlations are highly significant (p<0.001); large correlations |*r_s_*|≥0.5 are printed bold; due to missing values of some variables, the number of observations used for the calculations was 314≤n≤381; correlation between EQ VAS score and EQ-5D index was *r_s_*=0.69 (p<0.001); WHOQOL-BREF, World Health Organization Quality of Life-Bref questionnaire; BAI, Beck Anxiety Inventory; BDI-II, Beck Depression Inventory; BSQ, Body Sensation Questionnaire, ACQ, Agoraphobic Cognitions Questionnaire; MIA, Mobility Inventory - Subscale Avoidance Alone; MIB, Mobility Inventory - Subscale Avoidance Accompanied.
