# Supplementary material for: Validity and responsiveness of the EQ-5D in assessing and valuing health status in patients with anxiety disorders
Source: Health Qual Life Outcomes. 2010 May 5;8:47. doi: 10.1186/1477-7525-8-47 (PMC2873595; doi:10.1186/1477-7525-8-47)
Supplement: Additional file 7 — Table S7. Comparison of responsiveness statistics for EQ-5D scores and scores of measures used for comparison by anchor of change defined by BAI score [file 1477-7525-8-47-S7.DOC]

Table S7. Comparison of responsiveness statistics for EQ-5D scores and scores of measures used for comparison by anchor of change defined by BAI score

| Statistics | Measures | Anchor of change defined by BAI score | | |
| --- | --- | --- | --- | --- |
|  |  | More anxiety  *(N=43)* | Constant anxiety  *(N=124)* | Less anxiety  *(N=83)* |
| *t*-statistics  (paired *t*-test) | EQ VAS | **-2.60** | -0.65 | **2.29** |
|  | EQ-5D index | **-3.41** | 1.34 | **4.11** |
|  | WHOQOL BREF mental | **-2.10** | 0.77 | **3.16** |
|  | BSQ | **2.18** | -1.61 | **-6.56** |
|  | ACQ | **3.35** | 0.99 | **-5.56** |
| Effect size | EQ VAS | -0.33 | -0.04 | 0.28 |
|  | EQ-5D index | **-0.99** | 0.07 | 0.39 |
|  | WHOQOL BREF mental | -0.24 | 0.04 | 0.34 |
|  | BSQ | 0.27 | -0.12 | -0.67 |
|  | ACQ | 0.48 | 0.06 | -0.47 |
| Standardized response mean | EQ VAS | -0.40 | -0.06 | 0.26 |
|  | EQ-5D index | -0.54 | 0.13 | 0.46 |
|  | WHOQOL BREF mental | -0.32 | 0.07 | 0.35 |
|  | BSQ | 0.33 | -0.14 | -0.72 |
|  | ACQ | 0.51 | 0.09 | -0.61 |

^a^ Significant *t*-statistics (*p* < 0.05) and large effect sizes/standardized response means (>|0.8|) are printed bold; BAI, Beck Anxiety Inventory; WHOQOL-BREF mental, World Health Organization Quality of Life-Bref questionnaire – mental domain score; BSQ, Body Sensation Questionnaire; ACQ, Agoraphobic Cognitions Questionnaire.
