# Supplementary material for: Validity and responsiveness of the EQ-5D in assessing and valuing health status in patients with anxiety disorders
Source: Health Qual Life Outcomes. 2010 May 5;8:47. doi: 10.1186/1477-7525-8-47 (PMC2873595; doi:10.1186/1477-7525-8-47)
Supplement: Additional file 8 — Table S8. Results of the regression models estimating meaningful difference scores for various measures according to the anchor defined by the BAI scorea, controlled for measure's baseline score, BDI-II score and age of respondent (N = 230)b [file 1477-7525-8-47-S8.DOC]

Table S8 Results of the regression models estimating meaningful difference scores for various measures according to the anchor defined by the BAI score^a^, controlled for measure’s baseline score, BDI-II score and age of respondent (N=230)^b^

| Independent variables  Dependent variable: difference score  of measure | Coefficient values (SE)  [Coefficient values for standardised dependent variables (SE)] | | | | |
| --- | --- | --- | --- | --- | --- |
|  | EQ VAS^c^ | EQ-5D index^c^ | WHOQOL BREF^c^ mental | BSQ^c^ | ACQ^c^ |
| Shift to less anxiety:  dummy variable 1=yes; 0=no | **4.93** (2.45)  [**0.28** (0.14)] | **0.07** (0.03)  [**0.29** (0.11)] | 3.31 (2.07)  [0.23 (0.14)] | **-0.20** (0.08)  [**-0.32** (0.12)] | **-0.23** (0.06)  [**-0.54** (0.13)] |
| Shift to more anxiety  dummy variable 1=yes; 0=no | **-6.68** (2.74)  [**-0.38** (0.15)] | **-0.15** (0.04)  [**-0.67** (0.19)] | **-7.10** (2.34)  [**-0.50** (0.16)] | **0.21** (0.09)  [**0.34** (0.14)] | **0.16** (0.07)  [**0.37** (0.17)] |
| Score at baseline | **-0.49** (0.07)  [**-0.57** (0.09)] | **-0.40** (0.07)  [**-0.47** (0.08)] | **-0.39** (0.11)  [**-0.52** (0.14)] | **-0.49** (0.06)  [**-0.57** (0.07)] | **-0.25** (0.07)  [**-0.31** (0.08)] |
| Age of respondent (years) | **-0.26** (0.10)  [**-0.01** (0.01)] | **-0.00** (0.00)  [**-0.01** (0.00)] | -0.05 (0.07)  [-0.00 (0.01)] | **0.01** (0.00)  [**0.01** (0.00)] | 0.00 (0.00)  [0.00 (0.01)] |
| BDI-II score at baseline | -0.18 (0.14)  [-0.01 (0.01)] | -0.00 (0.00)  [-0.00 (0.01)] | -0.29 (0.21)  [-0.02 (0.01)] | **0.01** (0.00)  [**0.02** (0.01)] | 0.01 (0.00)  [0.01 (0.01)] |
| Constant | **45.69** (8.63)  [**0.76** (0.26)] | **0.40** (0.08)  [**0.53** (0.21)] | **28.56** (9.24)  [**0.57** (0.33)] | **-0.07** (0.14)  [**-0.74** (0.22)] | 0.03 (0.09)  [-0.30 (0.22)] |
| R² | 0.27 | 0.33 | 0.17 | 0.35 | 0.21 |
| R² increase due to inclusion of anchor defined by BAI score | 0.05^d^ | 0.10^d^ | 0.05^d^ | 0.05^d^ | 0.10^d^ |
| n | 225 | 220 | 228 | 230 | 230 |

^a^Patients whose psychopathology on the BAI decreased by more than 0.5 SD of the BAI baseline score from t0 to t1 were categorized as shifting to ‘less anxiety’ (*n*=43); patients whose BAI score increased by more than 0.5 SD were categorized as shifting to ‘more anxiety’ (*n*=83); all other patients were categorized as ‘constant anxiety’ (*n*=124); ^b^significant coefficients (p<0.05) are printed bold; ^c^ dependent variable is the difference score of the measure (score at t1 minus score at t0); ^d^significant R² increase (p<0.05) due to inclusion of anchor defined by BAI score; BAI, Beck Anxiety Inventory; BDI-II, Beck Depression Inventory; WHOQOL-BREF mental, World Health Organization Quality of Life-Bref questionnaire – mental domain score; BSQ, Body Sensation Questionnaire; ACQ, Agoraphobic Cognitions Questionnaire.
